# Los olvidados: Non-BRCA variants associated with Hereditary breast cancer in Mexican population

**DOI:** 10.1186/s13058-024-01957-9

**Published:** 2025-01-15

**Authors:** Dione Aguilar, María Lourdes Garza-Rodríguez, Carolina Elizabeth Muñiz-Garza, Fernando Alcorta Nuñez, Cynthia Mayte Villarreal-Garza, Oscar Vidal-Gutiérrez, Diana Cristina Pérez-Ibave, Carlos Horacio Burciaga-Flores

**Affiliations:** 1 Breast Cancer Center, Hospital Zambrano Hellion TecSalud, San Pedro Garza García, 64710 México, México; 2 Servicio de Oncología, Centro Universitario Contra el Cáncer (CUCC), Hospital Universitario “Dr. José Eleuterio González”, Universidad Autónoma de Nuevo León, 66451 Monterrey, Nuevo León México; 3 Facultad de Medicina, Universidad Autónoma de Nuevo León, Av. Francisco I. Madero S/N, Mitras Centro Monterrey, 64460 Monterrey, México; 4 Instituto Mexicano del Seguro Social (IMSS), Unidad Médica de Alta Especialidad, Hospital de Gineco Obstetricia (HGO), No. 23, 64000 Monterrey, Nuevo León México

**Keywords:** Non-*BRCA*, Breast cancer, Ovarian cancer, Pathogenic germline variants, Hereditary cancer

## Abstract

**Background:**

Hereditary predisposition to breast and ovarian cancer syndrome (HBOC) is a pathological condition with increased cancer risk, including breast (BC), ovarian cancer (OC), and others. HBOC pathogenesis is caused mainly by germline pathogenic variants (GPV) in *BRCA1* and *BRCA2* genes. However, other relevant genes are related to this syndrome diagnosis, prognosis, and treatment, including *TP53*, *PALB2*, *CHEK2*, *ATM*, etc. This study aimed to identify the prevalence of non-*BRCA* genes in HBOC patients of Northeast Mexico.

**Methods:**

This multicentric study included 1285 patients with HBOC diagnosis from four oncologic centers in northeast Mexico from 2016 to 2023. Genomic and clinical data were analyzed based on multi-gene panel results and electronic records of the medical geneticist consultation. For the data analysis of qualitative and quantitative variants, JASP statistical software (version 0.18.1) was used, taking p < 0.05 as a significant result.

**Results:**

We found that 32.7% of the patients had at least one GPV in non-*BRCA* genes. The five most frequent non-*BRCA* genes were *CHEK2*, *PALB2*, *MUTYH*, *CDKN2A*, and *ATM*. Among the group of non-*BRCA* genes, six are involved in the homologous repair pathway (HR), and three are related to DNA damage repair (DDR) pathways. In analyzing GPVs in molecular pathways, both have similar frequencies with no statistical difference for BC.

**Conclusion:**

Multi-gene testing implementation improves the detection of often overlooked genes related to HBOC pathogenesis and treatment. Non-*BRCA* GPVs in Northern Mexico correspond to one-third of the HBOC cases, including HR and DDR pathways genes that would be misdiagnosed if not tested. HR patient carriers are potential targets of iPARP therapies. The optimal approach to cancer treatment for non-*BRCA* mutation carriers warrants further investigation to develop newer therapies.

**Supplementary Information:**

The online version contains supplementary material available at 10.1186/s13058-024-01957-9.

## Introduction

Breast cancer (BC) and ovarian cancer (OC) have multifactorial etiology. Up to 10–15% of BC & OC are estimated to be hereditary[1, 2]. Hereditary predisposition to breast and ovarian cancer syndrome (HBOC) is a pathological condition caused mainly by germline pathogenic variants (GPV) in *BRCA1* and *BRCA2* genes, explaining 25–28% of BC and 40% of OC patients with a positive familial history [3]. *BRCA* carriers have a lifetime risk of 45–72% and 11–44% of developing BC and OC by age 70, respectively [2, 4, 5].

Thanks to newer technologies such as next-generation sequencing (NGS), several non-*BRCA* BC susceptibility genes have been identified, such as *ATM, CHEK2*, *PALB2*, *TP53*, etc. [6–15]. Identifying non-*BRCA* genes involved in the pathogenesis of HBOC improves diagnosis and gives the oncologist therapeutic options.

It is well-known that the germline genetic cancer susceptibility is heterogeneous [16]. The prevalence and spectrum of GPVs in HBOC cancer patients may vary across ethnicities [6, 17–20]. According to the National Comprehensive Cancer Network (NCCN) guidelines, the genetic test now incorporates 17 non-*BRCA* genes (*ATM*, *BARD1*, *BRIP1*, *CDH1*, *CHEK2*, *EPCAM*, *MSH2*, *MLH1*, *MSH6*, *NF1*, *PMS2*, *PALB2*, *PTEN*, *RAD51C*, *RAD51D*, *STK11*, and *TP53*) for a comprehensive analysis of the patients [21–23]. Globally, there is a tendency to include these genes in large cohorts to elucidate the pathogenicity of HBOC and address better recommendations for patients and their families [24, 25].

These genes have been selected as they are part of the DNA damage response (DDR) and repair mechanism; these are part of a pathway network protecting DNA integrity [26]. The main components in DDR are divided into the sensors of damage, the transducers of signals downstream, and the effectors that evaluate the cell’s fate by repairing the damage or executing apoptosis and immune destruction [27]. After the damage detection, one of six different repair pathways is mainly activated depending on the type of DNA damage: Homologous recombination (HR), non-homologous end joining (NHEJ), alternative end-joining (A-EJ) for double-strand breaks (DSBs), nucleotide excision repair (NER), for bulky DNA lesions; mismatch repair (MMR), for single-strand breaks (SSBs) and, base excision repair (BER) for oxidation, alkylation, deamination, and methylation damage [28]. DDR pathways do not play independently in the DNA repair machinery [26]. Genes encoding DNA response and repair pathways are generally mutated in cancer, causing genomic instability. This feature has been acknowledged by the scientific community, leading to the creation of targeted therapies for cancer treatment. One of the most prominent is the poly(ADP-ribose) polymerase PARP inhibitor that takes advantage of a deficit in the HR pathway to create a synthetic lethality that promotes cell death [26]. Treatments related to this feature are vital in hereditary cancer syndromes, where most genes are related to DNA repair mechanisms and can be used for a better outcome.

Mexico has limited information on the prevalence of non-*BRCA* genes related to HBOC [29, 30]. This study aimed to identify the prevalence of non-*BRCA* germline variants associated with HBOC in Northeast Mexico.

## Material and methods

### Patients and approval from the ethics committee

This multicenter study recruited 1285 patients from the Northern Mexican region from March 2016 to March 2023. All participant institutions have their oncologic service, including four public health institutions: The IMSS (Instituto Mexicano del Seguro Social), ISSSTE (Instituto de Seguridad y Servicios Sociales de los Trabajadores del Estado), Hospital Regional Materno Infantil, and Hospital Universitario (HU)” Dr. Jose Eleuterio González”; and two private institutions: Oncare Clinical Center, and Breast Cancer Center-Tec Salud.

Patients with a cancer diagnosis were evaluated in one of the participant centers and referred by a medical oncologist for a genetic evaluation due to the age of diagnosis and or family history of cancer. Self-referred patients came to consultation in the HU prevention clinic based on cancer personal or family history.

After the medical geneticist evaluation, genetic testing was offered to patients who met NCCN criteria for any hereditary cancer syndrome[23, 31]. The NCCN Guidelines® is the recognized standard for clinical direction and policy in cancer care. It is the most thorough and frequently updated clinical practice guidelines available in any area of medicine[23].

Patients with a clinical diagnosis of HBOC were invited to participate in the study and signed an informed consent letter [23]. Patients who did not fulfill the NCCN criteria for HBOC or could not undergo genetic testing were excluded. However, all patients with clinical suspicions of hereditary cancer were enrolled in a screening program according to the suspected diagnosis.

The protocol was approved by the Institutional Ethics Committee of the University Hospital  “Dr. José Eleuterio González” (registration number ON18-00015).

### Clinical data

Data from patients was collected in oncology and genetic consultation, and from electronic clinical records, including sex, age of diagnosis, cancer type, familial history, clinical stage, germline variants, etc.

### NGS multi-gene cancer panels and Sanger analysis

Genomic DNA was isolated from saliva or peripheral blood samples. External services multi-gene sequencing panels (from 7, 30, 35, and 84 genes) were performed. The 7 and 84 gene panels were made by Invitae Multi-Cancer Panel (©Invitae Corporation, San Francisco, CA, USA). The 30-gene panel was from Onco Life Test® (Life in Genomics®, Ciudad de México, México). We also included myRisk® Hereditary Cancer 35-gene panel (Myriad Genetics®, Salt Lake, UT, USA). Also, three patients had exome, and one had only Sanger sequencing. All variants were confirmed with Sanger sequencing. Panel selection was made based on the patient’s or institution’s availability and resources.

### Genetic counseling

Patients received face-to-face pre-test and post-test genetic counseling and laboratory reports from the leading geneticists (trained in oncogenetics) from each of the established hereditary cancer programs of Nuevo León México. Positive patients for genetic tests entered into an early detection and prevention program [20].

### Variant analysis and classification

According to the American College of Medical Genetics (ACMG) guideline [32], variants were classified as pathogenic, likely pathogenic, uncertain significance, likely benign, and benign. All variants were reviewed in ClinVar [33] and VarSome databases [34].

We compared the frequency of non-*BRCA* and *BRCA* against molecular pathway, cancer type, age of cancer diagnosis (< 40 years, 41–50, 51–60, and > 60 years), number of neoplasias, and the time lapse between tumors. We also studied the clinical stage and histologic type in BC (triple negative breast cancer (TNBC) and non-triple negative breast cancer (NTNBC)) and  pregnancy associated breast cancer (PABC). All analyses were made for all non-*BRCA* genes and individually for our cohort’s three most frequent genes (*CHEK2, PALB2, MUTYH*).

### Statistical analysis

Data analysis was reported as median and interquartile ranges, and the Mann–Whitney U test was used to compare all non-parametric quantitative variables between our groups (*BRCA* vs non-*BRCA*, PABC by the age of diagnosis, molecular pathways, and as between the groups and BC phenotype). Frequency, percentages, Fisher’s exact test, and *X*^2^ for qualitative variables were performed, including the frequency of *BRCA* and non-*BRCA* GPVs, neoplasias in patients and relatives, clinical stage, and association with multiple neoplasias. The JASP statistical software (version 0.18.1) was used for all statistical analyses, and statistical significance was set at p < 0.05.

## Results

### Clinicopathological data

The hereditary cancer programs evaluated 1285 patients, 1000 cancer patients, and 285 non-cancer patients. Among cancer patients, 327 (32.7%) were positive for GPVs related to HBOC: 317 (96.94%) carried one GPV, and 10 (3.05%) had two GPVs. The median age of diagnosis was 39 years (22–75); 318 (97.24%) were women, 11 (3.45%) with PABC, and 9 (2.75%) were men (Table 1).

**Table 1:** Demographic and clinical characteristics of the HBOC patients

Variable	n	%
Age at diagnosis		
Median (range)	39 (22–75)	
Sex		
Men	9	2.75
Women	318	97.24
Total	327	100.00
PABC	11	3.36
Cancer type		
BC unilateral	257	78.59
BC bilateral	33	10.09
Ovarian cancer	14	4.28
BC unilateral + Ovarian cancer	8	2.44
BC bilateral + Ovarian cancer	4	1.22
BC unilateral + Other	3	0.91
Prostate	2	0.61
Prostate + Other	2	0.61
BC bilateral + Other	1	0.30
Pancreas	1	0.30
Hemangioendothelioma + Cantú Syndrome	1	0.30
Melanoma	1	0.30

BC: Breast Cancer, PABC: Pregnancy Associated Breast Cancer

A positive family history of cancer was detected in 253 (77%) patients. Among the patients with a positive familial cancer history, second-degree relatives were the most affected, followed by first degree. Regarding cancer type in relatives, BC was by far the most frequent (53.7%), followed by OC (6.5%), gastric (5.3%), and prostate (5.1%) (Table 2).

**Table 2:** Family history of cancer

Family history of cancer			
	Yes (n = 253)		
	No (n = 71)		
	Unknown (n = 3)		
	First degree	Second degree	Third degree
Brain	2	3	0
Breast	130	158	133
Bladder	2	1	0
Cervical	8	12	5
Colon	6	25	5
Gastric	8	29	5
Head & Neck	3	10	4
Kidney	3	2	1
Leukemia	5	5	5
Liver	4	4	2
Lung	9	5	2
Lymphoma	1	0	4
Melanoma	6	1	2
Ovarian	23	18	10
Pancreas	12	18	6
Prostate	15	22	3
Thymus	0	1	0
Thyroid	1	2	0
Unknown	12	18	13

Most patients, 247 (75.53%), were referred from oncologic centers, and 70 (21.40%) were self-referred. The vast majority, 306 (93.57%) of our patients had BC diagnosis, and seven (2.14%) patients had non-HBOC-related neoplasias (cervical, lung, endometrial, colon, hemangioendothelioma, renal, and lymphoma) (Table 1).

We determined the risk factor of carrying a *BRCA* or non-*BRCA* GPV depending on the age of diagnosis. We found that by each year, there is a 1.029 times increase in the probability in favor of carrying a non-*BRCA* variant (OR = 1.029, 95% CI 1.003–1.055) and by each ten-year interval of 1.33. (OR = 1.33, 95% CI 1.035–1.724).

Most analyzed individuals had BC, unilateral in 268 (81.95%) and bilateral in 38 (11.62%). For the 145 TNBC analyzed cases, we observed a significantly higher frequency of *BRCA* GPVs than non-carrier patients (P-value < 0.001). Also, we did not find a difference in the age of diagnosis in TNBC patients versus other BC phenotypes.

In our cohort, BC was mainly found in the intermediate clinical stage, 200 (61.16%), followed by earlier stage 33 (10.09%), and 14 (4.28%) in an advanced stage, with 80 (24.46%) missing data. There was no statistical difference between the clinical stage and *BRCA* status.

There were 52 (15.90%) cases with more than one cancer (46 with two and 5 with three primary tumors). The median time lapse between the first and second cancer was 60 months and 48 months between the second and third diagnosis. We found no statistical difference between patients with one or multiple primary tumors and the presence of any GPVs (*BRCA* or non-*BRCA*).

In the PABC subgroup (n = 11), the mean age of diagnosis was earlier (33.09 years) than in the whole sample (40.13 years) (p = 0.007). Patients in this group were younger than 36 years (24–43 years), with only one patient who was 43 years old at diagnosis.

### GPVs and molecular pathways analysis

Multi-gene panels were used to diagnose the 1285 patients, including panels from 7, 30, 35, and 84 genes (Additional file 1: Table S1). From 327 positive patients, 80 were analyzed by the 7-gene panel, 85 by the 30-gene panel, ten by the 35-gene panel, and 158 with the 84-gene panel. Also, three patients had exome, and one had only Sanger sequencing. All variants were confirmed with Sanger sequencing.

**Fig. 1:**
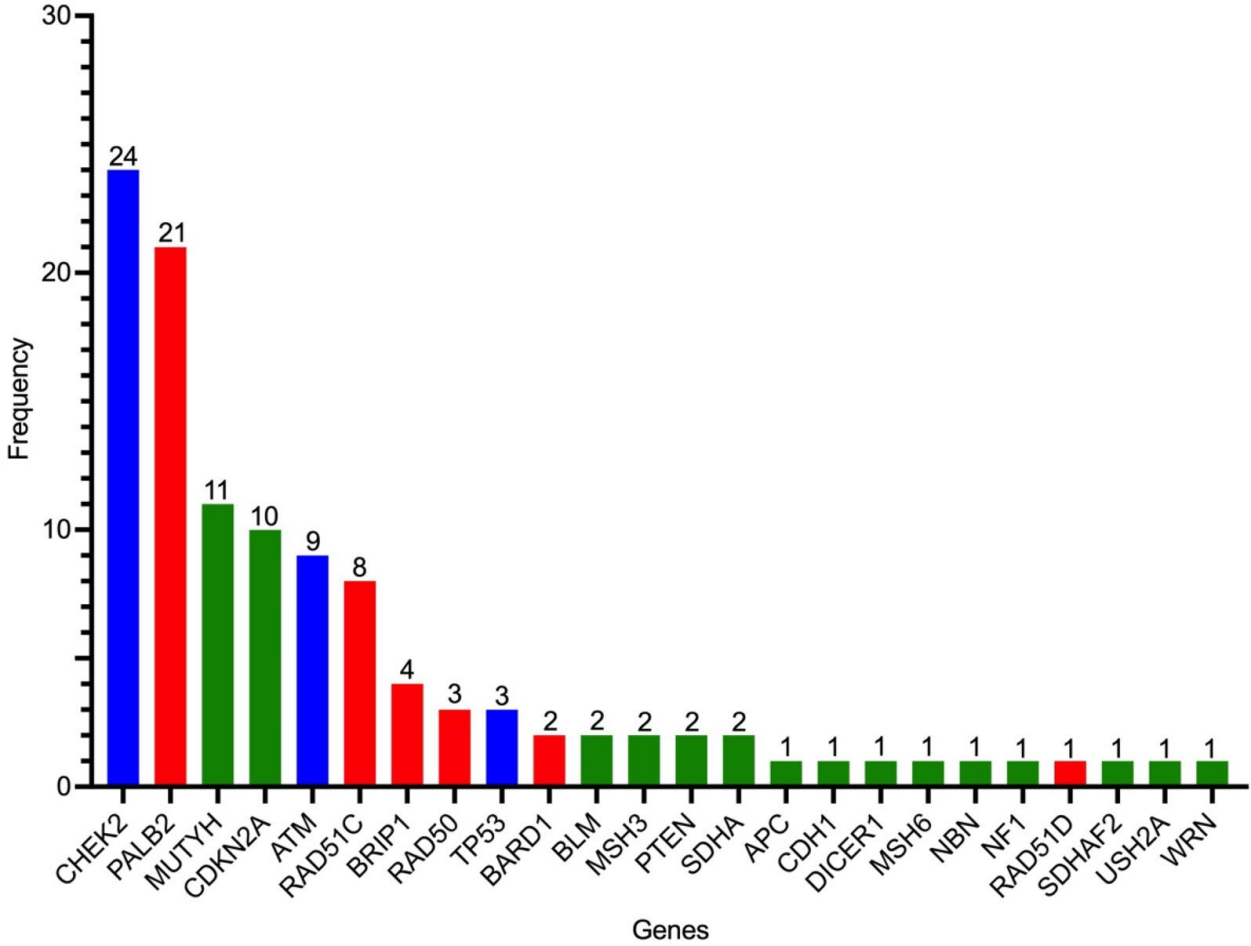
Frequency of Non-BRCA genes. Specific pathways are colored: HR genes are red, DDR genes are blue, and the remaining genes are green

*BRCA* GPVs were the most frequent among our cohort, with *BRCA1* accounting for 159 (47.18%) and *BRCA2* for 65 (19.28%). There were 113 GPVs (33.53%) in 24 non-*BRCA* genes. The five most frequent non-*BRCA* genes were *CHEK2* (24, 21.24%), *PALB2* (21, 18.58%), *MUTYH* (11, 9.73%), *CDKN2A* (10, 8.85%), and *ATM* (9, 7.96%). Among the group of non-*BRCA* genes, six were involved in the HR pathway, and three were related to the DDR pathway (Fig. 1).

The highest mutation rate for specific GPVs was *CHEK2* with c.707 T > C in 21/24 cases, followed by *MUTYH* c.1187G > A in 6/11 cases, and *CDKN2A* c.146 T > C in 6/10 cases and *PALB2* c.2167_2168del in 5/21 cases, all of them found in non-related patients. In the subgroup of PABC, three of them (27.27%) had non-*BRCA* GPVs (*CHEK2*, *PALB2 & MUTYH*).

For molecular pathways, we compared the age of diagnosis vs three groups (DDR, HR-*BRCA*, and HR non-*BRCA*), and we found a significant difference of p = 0.044. For the HR pathway divided into HR-*BRCA* (n = 222) and HR non-*BRCA* genes (n = 38), we found that HR-*BRCA* patients debuted younger than HR non-*BRCA* p = 0.041.

Regarding non-*BRCA* molecular pathways and cancer type, we analyzed BC (n = 72), HR (n = 38 53%), and DDR (n = 34 47%), as it was the most frequent neoplasia and we found no statistical difference between groups (p = 0.724).

In the individual analysis for the most frequent non-*BRCA* genes (*CHEK2*, *PALB2 & MUTYH*), there was an association between NTNBC and *CHEK2* carriers (p = 0.001).

## Discussion

Due to the implementation of multi-gene panels, the association of GPVs and HBOC has grown beyond *BRCA* genes. Unfortunately, in the oncology practice, non-*BRCA* genes are often forgotten [18]. Our study used various panels depending on the cost of panels and the sponsor (patient pay, foundations, donations, and investigation protocols). Most of our patients had broad gene panels (75%). Among our sample, 23.7% were analyzed by a 7-gene panel, which covered at least all high penetrance genes included in NCCN guidelines [23]. However, negative patients to the 7-gene panel could have a positive result if moderate penetrance genes were included. Further analysis of this population must be done to search for the detection rate. Also, three patients were included in exome analysis using a research protocol, and one patient was analyzed with Sanger because family GPV had already been detected.

Family history is still one of the main signs of suspected hereditary cancer; in our cohort, ¾ of our population had a positive family history of cancer. Interestingly, most patients had second-degree relatives. This is relevant as general knowledge focuses on the nuclear family, even when guidelines include second and third relatives supporting HSC suspicion. Also, the types of cancer found in relatives are according to expected in HBOC, with BC and OC being high in frequency. We found eight first and second-degree relatives of *PALB2* patients with prostate cancer, not generally associated with this neoplasia but has been reported with a more aggressive and lethal form of prostatic cancer [35]. For *CDKN2A,* a gene known for an increased risk of pancreatic cancer and melanoma [36], we found only a second-degree relative with pancreatic cancer. Lastly, for *MUTYH* relatives, we found only one second-degree relative with gastric cancer. As said before, *MUTYH* is known to increase the risk of gastrointestinal tumors [37]. There were 43 relatives with unknown cancers; when asked patients about this situation, they said that affected members tend to be silent on the topic as they prefer to hide the information due to shame or fear.

NCCN guidelines for Genetic/Familial High-Risk Assessment: Breast, Ovarian, and Pancreatic in the genetic testing section have changed since the start of the study in 2016; NCCN expanded the age of diagnosis for TNBC to include patients over 60 years in 2023 [22]. Our cohort range of testing went from 22 to 75 years (median 39 years). With the updated criteria in 2023, we found 181 patients with GPVs, of which 19 (10.49%) were ≥ 60 years old, a considerable number of cases that would be lost with previous recommendations.

Our detection rate for GPVs (*BRCA* and non-*BRCA*) was 32.7%, similar to other Latin and Mexican series with 10–30% detection rates [19, 29, 38]. As in our cohort, patients older than 60 only account for 5.8% of GPVs, a population recently included in guidelines that can increase detection rates.

In our cohort, 2.2% of the patients had non-HBOC-related neoplasias. These patients had synchronic or metachronic neoplasias in other organs not commonly associated with the HBOC spectrum, including cervical, lung, endometrial, colon, hemangioendothelioma, renal, and lymphoma. Other studies also report unusual phenotypes in patients with hereditary cancer syndromes like Lynch and Banayan Riley Ruvalcaba [39–41]. Among these patients, there were two cases with non-*BRCA* GPVs, one case with synchronic prostate and renal cancer with a *MUTYH* GPV, and a case of synchronic BC and lung cancer with an *ATM* GPV. It has been reported that monoallelic *MUTYH* GPVs are related to renal neoplasia [42]. *ATM* GPVs can also increase the risk of lung adenocarcinoma [43]. This association needs to be validated with larger patient cohorts. Our results support the idea that there is no complete understanding of the non-classic spectrum of neoplasias associated with HBOC, reflecting the complexity of the syndrome.

There were no differences in the clinical stage of BC in our cohort compared with other reports for the Mexican population, where almost half of the patients are diagnosed in locally advanced stages [44].

There was an association between TNBC and *BRCA* and NTNBC patients with *CHEK2* GPVs. Other reports associate *TP53* with HER2 + BC [45], *CHEK2* with Luminal B [46], and *BRCA* with TNBC [47], both concordant with our results. *CHEK2* is considered a highly penetrant gene with a moderate risk for BC (20–40%) in both monoallelic and biallelic states [48, 49]. There was insufficient statistical power to associate genotype–phenotype in the less frequent non-*BRCA*; further analysis with increased cases may address this topic.

We found 33.53% of non-*BRCA* GPVs, higher than other series, including Asian, Spain, and Latin American populations [50–53], with a range of detection between 4–12%. The genetic admixture of the northern Mexican population, the selection of patients, and the Latino population reporting higher *CHEK2* c.707C > T GPV could explain why we had higher detection rates than other reports [54].

Regarding the frequency of the non-*BRCA* gene, we found that *CHEK2* was the most frequent, followed by *PALB2*, *MUTYH*, *CDKN2A*, and *ATM*. Other studies in different populations report *PALB2* as the most frequent non-*BRCA* gene, followed by *CHEK2*, *ATM* and/or *TP53*. In Latin populations, *CHEK2* GPVs are reported as the most frequent non-*BRCA* GPVs, addressing the 707 T > C with a possible founder effect [54]. Unfortunately, none of these studies analyzed *MUTYH* or *CDKN2A* GPVs, even when international guidelines include them in HBOC patient testing.

Even though there is conflicting evidence of the potential pathogenicity of *MUTYH* in a monoallelic state, there has been an association with BC not only in the development of the disease but also in the characteristics of the tumor, showing more aggressive behavior and diversity [37]. Also, in various cohorts of BC patients, monoallelic *MUTYH* GPVs are always the most frequent findings [55–57], even suggesting more strict surveillance [57]. For *CDKN2A* GPVs, recent evidence has been associated with an increased risk of BC development (OR: 3.35, 95% CI: 1.43–7.75). In our cohort, *MUTYH* and *CDKN2A* had a higher frequency than *ATM* or *TP53*. Our findings explain the importance of population-specific analysis and multi-gene panels in understanding HBOC physiopathology.

From the patients with two or more neoplasias, we found that the time-lapse between cancer was no different for the GPVs in *BRCA* or non-*BRCA* carriers, no matter the penetrance of the genes. These results highlight the idea that HBOC is a complex diagnosis with similar behavior regardless of the etiology. Based on our results, we recommend that non-*BRCA* patients should have the same strict surveillance as *BRCA* patients.

In the subgroup of PABC, we found a difference in the age of diagnosis compared to the whole sample. This has been reported in other studies in pregnant women with *BRCA* GPVs, who have a younger presentation than non-carriers [58]. Also, some studies found a higher risk of cancer with each pregnancy [59]; meanwhile, other studies have observed that only *BRCA2* increases the risk of cancer before 50 years [60]. Our study found six patients with *BRCA2*, two with *BRCA1*, and three with non-*BRCA* genes. However, there is no clear evidence of the role of non-*BRCA* genes in the development of PABC or other neoplasias. We need to increase our population to analyze the effect in this subgroup.

In the analysis of GPVs in molecular pathways, we found that age of appearance and tumor type were relevant, as patients younger than 40 years were associated with *BRCA* GPVs, and patients with OC had a higher chance of carrying an HR gene GPV. Also, patients with GPV in DDR genes had a higher risk of developing BC and having cancer between 41–50 years. This is important because some tumors in the HBOC spectrum have molecular features for *BRCA* and non-*BRCA* genes related to an HR pathway deficiency that can be exploited as therapeutic targets like PARP inhibitors. Although PARP inhibitors are mainly used in *BRCA* + tumors, recent studies also suggest high responses in non-*BRCA* mutated cells, including genes like *ATM, ATR, RAD51*, and *BARD1* that are related to genomic instability generation and indicative of homologous recombination deficiency (HRD). The fact that in our study, 31.85% of the non-*BRCA* carriers had DDR GPVs like *ATM* opens the possibility of applying PARP inhibitors as monotherapy or, in combination with *ATM* inhibitors, can improve the prognosis of these patients. The phase I clinical trial (NCT02588105) assessed that the use of *ATM* inhibitors as monotherapy had low antitumor effects, while pre-clinical studies in cell lines showed that the combination of the novel *ATM* inhibitor AZD0156 in combination with iPARP leads to an increase in DNA double-strand break signaling, cell-cycle arrest, and apoptosis [27].

Exploring genes outside the most commonly known raises awareness of the frequency of these often overlooked potential targets to develop and implement new targeted therapies, such as monotherapy or drug combinations, to enhance actual treatments and find ways to bypass drug resistance and cancer progression.

## Conclusions

Non-*BRCA* GPVs in Northern Mexico correspond to one-third of the BC and OC cases, including HR and DDR pathways genes. HR patient carriers are potential targets of iPARP therapies. An optimal approach to cancer treatment for non-*BRCA* mutation carriers warrants further investigation. This project reinforces that multi-gene panels should be implemented as a standard of care in HBOC to ensure a complete diagnosis in hereditary cancer patients and improve surveillance strategies in non-*BRCA* patients.

## Supplementary Information

**Additional file 1**: Multi-gene panels analyzed.

## Data Availability

No datasets were generated or analysed during the current study.

## Abbreviations

HBOC: Hereditary breast ovary cancer
BC: Breast cancer
OC: Ovary cancer
GPV: Germline pathogenic variant
BRCA1: Breast Cancer 1
BRCA2: Breast Cancer 2
HR: Homologous repair pathway
DDR: DNA damage repair
ATM: ATM Serine/Threonine Kinase
BARD1: BRCA1 Associated RING Domain 1
BRIP1: BRCA1 Interacting Helicase 1
CDH1: Cadherin 1
CHEK2: Checkpoint Kinase 2
EPCAM: Epithelial Cell Adhesion Molecule
MSH2: MutS Homolog 2
MLH1: MutL Homolog 1
MSH6: MutS Homolog 6
NF1: Neurofibromin 1
PMS2: PMS1 Homolog 2, Mismatch Repair System Component
PALB2: Partner And Localizer Of BRCA2
PTEN: Phosphatase And Tensin Homolog
RAD51C: RAD51 Paralog C
RAD51D: RAD51 Paralog D
STK11: Serine/Threonine Kinase 11
TP53: Tumor Protein P53
IMSS: Instituto Mexicano del Seguro Social
ISSSTE: Instituto de Seguridad y Servicios Sociales de los Trabajadores del Estado
HU: Hospital Universitario
NCCN: National Comprehensive Cancer Network
NGS: Next generation sequencing
TNBC: Triple-negative breast cancer
NTNBC: Non-triple-negative breast cancer
iPARP: Poly ADP-ribose polymerase inhibitors
