# Supplementary material for: Los olvidados: Non-BRCA variants associated with Hereditary breast cancer in Mexican population
Source: Breast Cancer Res. 2025 Jan 15;27:7. doi: 10.1186/s13058-024-01957-9 (PMC11737022; doi:10.1186/s13058-024-01957-9)
Supplement: Supplementary file 1 — Additional file 1: Multi-gene panels analyzed. [file 13058_2024_1957_MOESM1_ESM.docx]

Table S1 Multi-gene panels analyzed

| Gene Panel | Provider | Genes analyzed | Patients analyzed |
| --- | --- | --- | --- |
| Hereditary Breast Cancer Panel | Invitae | BRCA1, BRCA2, CDH1, PALB2, PTEN, STK11, TP53 | 80 |
| Multi-Cancer Panel | Invitae | AIP, ALK, APC, ATM, AXIN2, BAP1, BARD1, BLM, BMPR1A, BRCA1, BRCA2, BRIP1, CASR, CDC73, CDH1, CDK4, CDKN1B, CDKN2A (p14ARF), CDKN2A (p16INK4a), CEBPA, CHEK2, CTNNA1, DICER1, DIS3L2, EGFR, EPCAM, FH, FLCN, GATA2, GPC3, GREM1, HOXB13, HRAS, KIT, MAX, MEN1, MET, MITF, MLH1, MSH2, MSH3, MSH6, MUTYH, NBN, NF1, NF2, NTHL1, PALB2, PDGFRA, PHOX2B, PMS2, POLD1, POLE, POT1, PRKAR1A, PTCH1, PTEN, RAD50, RAD51C, RAD51D, RB1, RECQL4, RET, SDHA, SDHAF2, SDHB, SDHC, SDHD, SMAD4, SMARCA4, SMARCB1, SMARCE1, STK11, SUFU, TERC, TERT, TMEM127, TP53, TSC1, TSC2, VHL, WRN, WT1 | 158 |
| Onco Life Test | Life in Genomics | APC, ATM, BAP1, BARD1, BMPR1, BRCA1, BRCA2, BRIP1, CDH1, CDK4, CDKN2A (p14ARF), CDKN2A (p16INK4a), CHEK2, EPCAM, GREM1, MITF, MLH1, MSH2, MSH6, MUTYH, NBN, PALB2, PMS2, POLD1, POLE, PTEN, RAD51C, RAD51D,  SMAD4, STK11, TP53 | 85 |
| MyRisk | Myriard | APC, ATM, AXIN2, BARD1, BMPR1A, BRCA1, BRCA2, BRIP1, CDH1, CDK4, CDKN2A, CHEK2, EPCAM, HOXB13, GALNT12, GREM1, MLH1, MSH2, MSH3, MSH6, MUTYH, NBN, NTHL1, PALB2, PMS2, PTEN, POLE, POLD1 RAD51C, RAD51D, RNF43, RPS20, SMAD4, STK11, TP53 | 10 |
| Exome | 3Billion | Exome | 3 |
| Sanger | In House | BRCA1 | 1 |
